# From Toxicity to Selectivity: Coculture of the Fluorescent Tumor and Non-Tumor Lung Cells and High-Throughput Screening of Anticancer Compounds

**DOI:** 10.3389/fphar.2021.713103

**Published:** 2021-10-11

**Authors:** D.A. Skvortsov, M.A. Kalinina, I.V. Zhirkina, L.A. Vasilyeva, Y.A. Ivanenkov, P.V. Sergiev, O.A. Dontsova

**Affiliations:** ^1^ Chemistry Department, Faculty of Bioengineering and Bioinformatics, Lomonosov Moscow State University, Moscow, Russia; ^2^ Faculty of Biology and Biotechnologies, Higher School of Economics, Moscow, Russia; ^3^ Skolkovo Institute of Science and Technology, Moscow, Russia; ^4^ Institute of Biochemistry and Genetics Russian Academy of Science (IBG RAS), Ufa Scientific Centre, Ufa, Russia; ^5^ Shemyakin‐Ovchinnikov Institute of Bioorganic Chemistry, Moscow, Russia

**Keywords:** phenotypic screening, anticancer compounds, cytotoxicity, high-throughput, fluorescence detection, lung cancer model

## Abstract

For the search of anticancer compounds in modern large chemical libraries, new approaches are of great importance. Cocultivation of the cells of tumor and non-tumor etiology may reveal specific action of chemicals on cancer cells and also take into account some effects of the tumor cell’s microenvironment. The fluorescent cell cocultivation test (FCCT) has been developed for screening of substances that are selectively cytotoxic on cancerous cells. It is based on the mixed culture of lung carcinoma cells A549’_EGFP and noncancerous fibroblasts of lung VA13_Kat, expressing different fluorescent proteins. Analysis of the cells was performed with the high-resolution scanner to increase the detection rate. The combination of cocultivation of cells with scanning of fluorescence reduces the experimental protocol to three steps: cells seeding, addition of the substance, and signal detection. The FCCT analysis does not disturb the cells and is compatible with other cell-targeted assays. The suggested method has been adapted for a high-throughput format and applied for screening of 2,491 compounds. Three compounds were revealed to be reproducibly selective in the FCCT although they were invisible in cytotoxicity tests in individual lines. Six structurally diverse indole, coumarin, sulfonylthiazol, and rifampicin derivatives were found and confirmed with an independent assay (MTT) to be selectively cytotoxic to cancer cells in the studied model.

## Introduction

Exploring low molecular weight compounds for the discovery of novel molecules for chemotherapy is in the list of priority fields for cancer drug development of the Lancet Oncology Commission (Jaffee et al., 2017). The modern chemical libraries contain thousands of LMW (low molecular weight) compounds, and to analyze them, the applications of new methods of screening and appropriate models of tumors are of great importance. Moreover, it is desirable to reveal the specificity of the action of chemicals on cancer cells at the initial stages of drug development (Cagan, 2016).

There are two main approaches for the primary selection of the potential anticancer compounds: target-based and phenotypic searches. The first approach can be applied when a pathway that is deregulated in cancer is known. In this case, discovered screening compounds are targeted to one specific molecular mechanism (for review, see Seebacher et al., 2019). The addressed influence of a substance on this pathway may not reflect its overall biological activity. Potential anticancer compounds with other targets may be skipped in such searches. Despite some drawbacks, this approach has proven to be effective for cancer drug development (Yaish et al., 1988; Levitzki and Klein, 2019). Addressed screening of novel agents is especially favorable when a new molecular target is available. The second approach is phenotype based; it is focused on the selection of substances that alter parameters of the cell or organism in the desired way. The preannounced molecular mechanism of the disease is not required, and the search of the active compounds is not limited to known targets. However, the complexity of the approach is that the mechanism of action of the discovered compounds has to be determined after the phenotypic search.

Phenotypic screening has already been used for cancer drug screening for several decades (Carmichael et al., 1987). Since the goal of chemotherapy is the elimination of the cancer cells, the simplest desired phenotype for cancer drug selection is the death of tumor cells (Ediriweera et al., 2019). The cytotoxicity data obtained in the tests allow determining the selectivity of the compound action *in vitro*. The cytotoxicity evaluation can be performed in monocultures or cocultures in the 2D or 3D growth mode of the cell lines. The biological activity of a substance assessed on the 2D monoculture of adhesive cancer cells may not sufficiently reflect the activity of the same substance *in vivo* (Jo et al., 2018). More complex 3D cultivation allows one to take into account the concentration gradient of a substance in a solid tumor and microenvironment features that may include immune or stromal cell interactions (for review see (Jo et al., 2018; Kitaeva et al., 2020)). Three-dimensional tumor models based on the mixed cultures can be used to evaluate the specificity of action as an initial parameter of a compound. Despite many advantages of 3D models (Miki et al., 2012; Jo et al., 2018), the complexity of their cultivation and reproducibility, high cost, and limited performance hinder their routine usage in screening (Stock et al., 2016). That is why the cells growing in the monolayer are still widely applied for screenings (Shoemaker, 2006; Seebacher et al., 2019). Novel models, conjoining mixed cultures, and simplicity of 2D models may be useful for selectivity-based screening of anticancer compounds.

Cocultivation of various cell lines is used mainly for investigations of normal cell interactions and the tumor microenvironment. There are cell growth approaches, based on conditioned media usage, cultivation of cells through a membrane with micropores, and mixed cultures. Mixtures of isogenic cell lines can be used for probing multidrug resistance (Brimacombe et al., 2009; Windt et al., 2019). Cells of different origins, e.g., tumor and stroma, are widely used for the study of cell–cell interactions (for review see (Miki et al., 2012; Jo et al., 2018)). Even the cocultivation of the cells of different organisms is useful for the detection of viruses (Leland and Ginocchio, 2007). The cocultures of different origins from one organism are fashionable to model tissues (Baker, 2011) and investigate cytotoxic effects on cell ensembles (Alfaro-Moreno et al., 2008). Thus, the treatment with 17β-estradiol inhibits the proliferation of the MCF-7 tumor cell line cocultivated with noncancerous MCF10A, while this effect was not observed in the monoculture of MCF-7 cells (Spink et al., 2006). Growth of lung adenocarcinoma cells A549 together with SV-80 fibroblasts increases the survival of the tumor cells compared to that of monoculture, where expression of Ki-67 appears in A549, and the level of markers of mesenchymal transition changes (Amann et al., 2014). Growth of macrophages with A549 increases the production of cytokines by macrophages, promoting tumor growth (Muller-Quernheim et al., 2012). Cocultivation can also be applied in screening (Miki et al., 2012; Brimacombe et al., 2009), but it is rarely used in practice. Thus, the displacement of normal cells by rapidly growing tumor lines was proposed as a tumor model for drug search but was not applied in screening (El Debs et al., 2011).

Lung cancer is one of the most common causes of tumor lesions and related deaths in the world, according to the WHO data (Ferlay et al., 2015). Therefore, lung tumor cells are an actual target for the search for new anticancer substances.

In this work, we propose the mixed culture of lung carcinoma cells A549′ and noncancerous fibroblasts of the lung cell line VA13 to search for substances with selective toxicity against cancerous cells. The coculture is the simplest *in vitro* tumor model for the tumor cell’s microenvironment. The fluorescent cell cocultivation cytotoxicity test (FCCT) based on 2D cocultivation of cell lines labeled with fluorescent proteins was developed for high-throughput application: low expenses and enhanced performance. It was utilized for the screening of 2,491 structurally diverse substances. Several identified compounds have supported this approach for screening of selective substances against cancer cells.

## Materials and Methods

### Cell Lines and Culture Conditions

Human cell lines A549′, VA13, and HEK293T were maintained in DMEM/F-12 media containing 10% FBS, 50 u/ml penicillin, and 0.05 mg/ml streptomycin (all products from Thermo Fisher Scientific, United States) at 37°С in 5% CO_2_. Medium F-12 (Paneco LLC, Russia) containing 10% fetal bovine serum, 50 U/ml penicillin, and 0.05 mg/ml streptomycin was used in the FCCT assay. A549′ is the fast growth subline of A549 adenocarcinoma cell lines; VA13 is the WI38 subline 2RA, immortalized lung fibroblasts, HEK293T is a highly transfectable derivative of human embryonic kidney 293 cells and contains the SV40 T-antigen. Cell cultures were genotyped by STR (Supplementary Figure S1 for A549’, VA13) and tested for the absence of mycoplasma.

### Construction of Cell Lines Stably Expressing Fluorescent Proteins

Vectors LeGO-iG2, LeGO-C2 (Addgene, United States), and LeGO-K2 (Kalinina et al., 2018) were used for integration of the genes of EGFP, mCherry, and Katushka2S, respectively. For the virus’s production, the HEK293T cell line was used. Five million cells were seeded in 10-cm plates, and on the next day, the subconfluent cells were transfected with 20 µg of the plasmid LeGO-iG2, LeGO-C2, or LeGO-K2 per plate together with lentivirus helper constructs pMDLg/pRRE (10 µg), pRSV-REV (5 µg), and pMD2.G (2 µg). Transfections were performed by the calcium phosphate method according to the protocol from http://www.lentigo-vectors.de/protocols.htm. After 24 and 48 h, the medium was collected and filtered through a 0.22 µm filter (Millipore, United States). Transduction of VA13 and A549′ cells was performed in accordance with the LeGO system manufacturer protocol. In brief, 100,000 cells were seeded per well in a six-well plate in media containing 10% FBS, 50u/ml penicillin, and 0.05 mg/ml streptomycin (all products from Thermo Fisher Scientific, United States). After 15 h of the cultivation, the medium was substituted for the same one with lentiviruses containing the corresponding construct and polybrene (8 μg/ml). Virus solution quantities were selected for more than 90% infection of the cells. The plate was centrifuged for 1 h at 1,000g and 24°C, followed by 24 h of incubation at 37°С and 5% СО_2_. Then the medium was replaced with complete DMEM/F-12.

### Microscopy

Microscopic control of cells was performed using the fluorescence microscope EVOS FL Cell Imaging System. EGFP was detected in the GFP channel, and Katushka2S, in the RFP channel. In the images of VA13 and A549’ cocultures, the EGFP is shown in the green channel, and Katushka2S, in the red one. An example of fluorescent cells is shown in Supplementary Figure S2.

### Chemicals

The library consists of 2,491 structurally diverse natural substances, and their derivatives was obtained from InterBioScreen. Sorafenib was obtained from ChemRar. Bortezomib, cisplatin, and 5F-uracil were produced by Teva. Nocodazole and cycloheximide were purchased from Sigma.

### Fluorescent Cell Cocultivation Test Assay

Polycarbonate 96-well (Greiner #677180 or Eppendorf #0030730.127) and 384-well (Greiner #781182) plates were used for cell growth and fluorescence detection. Cytotoxicity evaluation of known drugs was performed in triplicate in 96-well plates. High-throughput screening was performed in 384-well plates, and an auto-pipette station Janus (PerkinElmer) was used. 1,600 and 3,200 cells corresponding to А549’_EGFP/VA13_Kat were seeded per well of a 96-well plate in 100 µL of F12 media. A total of 400 and 800 cells corresponding to А549’_EGFP/VA13_Kat were seeded per well of a 384-well plate in 40 µL of F12 media. Cells were grown for 16–18 h without treatment for attachment to the plate surface.

Then, 8.5 or 10 mg/ml of the compound’s stock solutions was diluted by 1:100 in F12 media in microplates and centrifuged at 2000 g for 5 min. Up to 40 µL of the solutions of the compounds (2–40 µL) and media was added to the wells. Then the cells were incubated for 72 h at 37°С and 5% СО_2_ and scanned with a TYPHOON FLA950 (GE Healthcare). The maximum 10-micron resolution of the scanner was used. The following lasers and settings were used for imaging of the cells: the 473 nm laser (with the voltage adjusted to 600 V) and the 520–540 nm emission filter were used for eGFP; the 560–580 nm filter and the 532 nm laser (attuned to 500 and 750 V correspondingly) were used for mCherry; and the 635 nm laser (850 V) and the ≥665 nm filter were used to image Katushka2S.

Processing of plate images was carried out using the ImageJ editor (https://imagej.nih.gov/ij/). The image of the well was duplicated, and one copy was subjected to the Gaussian blur filter with a radius of 10 pixels for a 384-well plate. The obtained pseudo-background image was subtracted pixel-wise from the initial image. Less than zero pixels was equated to zero (Kalinina et al., 2018). Intensity percentages were calculated in processed images of the wells as described in (Guzman et al., 2014). Automatic calculation in a single-replicate assay could lead to data distortion; therefore, the selected compounds were additionally checked “by eyes.” The data on the survival of A549’_EGFP cells were normalized to the VA13_Kat survival to calculate the selectivity. We consider active substances that have at least two-fold selectivity in two or more serial dilutions of tested compounds.

### Statistical Test

The data for statistical tests were analyzed and visualized using Rstudio software version 1.3.959 and the ggplot2 package (Wickham, 2016). The difference between CC50 values was assessed and visualized by the ggsignif package (Ahlmann-Eltze and Patil, 2021). CC50 values were compared by the Wilcoxon test.

### Mosmann Assay

VA13 4,000 cells or A549’ 2,500 cells per well were seeded in a 96-well plate in DMEM-F12 media containing 10% fetal bovine serum, 50 U/ml penicillin, and 0.05 mg/ml streptomycin. After 18 h of growth, an investigated substance diluted in the culture medium was added to cells. The cells with the added compound were incubated for 72 h at 37°С and 5% СО_2_. Then, the MTT reagent (Paneco LLC, Russia) was added to cells up to the final concentration of 0.5 g/L (10X stock solution in PBS was used) and incubated for 2 h at 37°C in the incubator, under an atmosphere of 5% CO_2_. The MTT solution was then discarded, and 140 µL of DMSO (PharmaMed LLC, Russia) was added. The plates were swayed 10 min on a shaker (80 rpm) to solubilize the formazan. The absorbance was measured using a microplate reader (VICTOR X5 Light Plate Reader, PerkinElmer, United States) at a wavelength of 565 nm. The results were used to construct a dose–response graph and calculate the IC50/CC50 value (GraphPad Software, Inc., San Diego, CA).

### Calcein AM Assay

The calcein AM assay was based on the live and dead assay protocol (ThermoFisher). 2,500 cells per well for the A549’ cell line or 4,000 cells per well for the VA-13 cell line were plated out in 140 µL of F12 media (Paneco LLC, Russia) in a 96-well plate and incubated in the 5% CO_2_ incubator for the first 16 h without treating. Then, 10 µL of media-DMSO solutions of tested substances was added to the cells (final DMSO concentrations in the media were 0.5% or less), and cells were treated for 72 h with 0.39–50 µM (eight dilutions) of our substances (triplicate each). After incubating with tested compounds, cells in the 96-well plate were rinsed with PBS and then incubated with 3 µM calcein AM (ThermoFisher) solution in PBS for 30 min. After incubation, cells were rinsed with PBS and then fluorescence was measured in a microplate fluorometer (VICTOR X5 Light Plate Reader, PerkinElmer, United States) with excitation at 485 nm and emission at 535 nm and scanned with a TYPHOON FLA950 (GE Healthcare). The maximum 10-micron resolution of the scanner was used. The following lasers and settings were used for imaging the cells: the 473 nm laser (with the voltage adjusted to 650 V) and the 520–540 nm emission filter. The results were used to construct a dose–response graph and to estimate the CC50 value (GraphPad Software, Inc., San Diego, CA).

## Results

Cocultivation of cells of malignant and non-malignant etiology originating from the same organ was proposed to search for selectively toxic compounds to tumor cells. The adenocarcinoma cell line A549’ and immortalized embryonic lung fibroblasts VA13 were selected to simulate lung cancer at the screening.

### Preparation and Imaging of the Fluorescent Cell Lines

**FIGURE 1:**
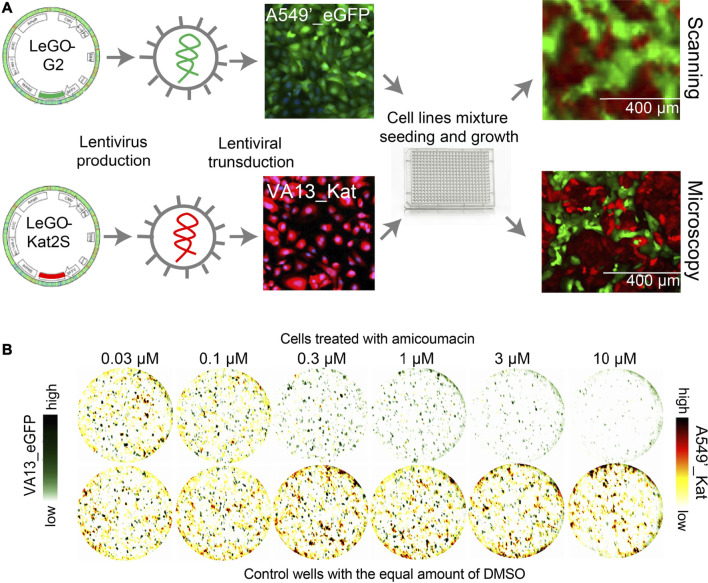
Preparation and imaging of fluorescent cell lines A549’_EGFP/VA13_Kat and VA13_EGFP/A549’_mCherry. **(A)** The scheme of labeling of the cell lines by lentiviral integration of fluorescent protein genes with examples of A549’_EGFP/VA13_Kat cells. **(B)** Images of mixed culture of A549’_EGFP/VA13_Kat cells obtained by scanning at a resolution of 10 µm** (top)** and by microscopy **(bottom)**. **(C)**. Images of the coculture of VA13 and A549′ derivative cell lines VA13_EGFP и A549’_mCherry. The images were obtained by superimposing scanned data in the channels of red and green fluorescent proteins. The upper row of imaged wells was treated with a series of dilutions of the known selective compound amicoumacin. VA13 cells (green channel) are alive much more than A549′ tumor cells at a concentration of amicumacin of 0.3–3 µM. As a reference, at the **bottom** row, there are demonstrated cells to which DMSO (drug solvent) was added at the appropriate concentrations.

The cells were modified by the lentiviral integration of fluorescent protein genes in their genomes. Fluorescent proteins EGFP (excitation maximum 488 nm, emission maximum 510 nm) and Katushka2S (excitation maximum 588 nm, emission maximum 633 nm) were selected for labeling to avoid overlapping of fluorescent spectra during imaging. The gene of green fluorescent protein EGFP was expressed in A549’_EGFP cells; the gene of far-red fluorescent protein Katushka2S was expressed in VA13_Kat cells as described earlier (Kalinina et al., 2018). The scheme of modification of A549’_EGFP and VA13_Kat lines and their growth in monocultures and cocultures are shown in Figure 1A.

The imaging of labeled cells was performed with a high-resolution scanner TyphoonFLA at the maximum resolution of 10 µm. This allows us to perform scanning of the 384-well plate in one channel within 15 min, which is faster than most microscopy-based solutions and improves the performance of the method. The price for a high rate is low resolution compared to that of microscopy, but enough for quantification of the cells. The growth of cells and their fluorescence were monitored microscopically. Images of the cell coculture obtained with a scanner and fluorescence microscope are shown in Figure 1B.

The assay may be based on other schemes of cell labeling with fluorescent proteins. There can be utilized vice versa labeled cell lines: A549’_Kat, which expressed Katushka2S and VA13_EGFP, labeled with EGFP (Supplementary Figure S2). The possibility of labeling cells with other fluorescent proteins was demonstrated by imaging the coculture of VA13_EGFP и A549’_mCherry. The images of this mixed culture without treatment and with amicoumacin treatment (that is selectively cytotoxic for these cell lines (Prokhorova et al., 2016)) are shown in Figure 1C.

### Cocultivation of A549’_EGFP and VA13_Kat Cells

The cell line VA13 has a slower growth rate compared to A549′ cells (Supplementary Figure S3,S4). In order to take this into account, the ratio 2:1 of the quantities of seeded VA13_Kat and A549’_EGFP cells was selected (tested ratios were between 1:3 and 3:1). Seeding density was 100 for VA13_Kat cells and 50 for A549’_EGFP cells per mm^2^ of a surface. Media F12 with a low riboflavin concentration for cell growth was chosen to reduce the background signal of green fluorescence (Kalinina et al., 2018). Under these conditions, cells of both lines attached to the surface overnight after seeding and reached a monolayer on the fourth day. Both cell lines grow as a mixture of initially seeded homogenously distributed cells, without a strong tendency to aggregate cells of the same type (Figure 1B, Supplementary Figure S4). Their growth close to the monolayer under competitive conditions allows us to assume that it would consider some of the microenvironment effects.

### The Evaluations of Cytotoxicity in Co-culture on a Panel of Known Drugs

The viability of cells in the mixture was measured with the suggested fluorescent cell cocultivation cytotoxicity test (FCCT). Two parameters of the high-resolution image can be used for the cell quantification. They can be 1) the total fluorescence intensity of all pixels exhibiting fluorescence, corresponding to the intensity of the fluorescence signal from cells in the well or 2) the total area of all pixels exhibiting fluorescence, corresponding to the area of the fluorescent cells in the well. Calculations based on the cell fluorescence intensity better reflect the density of the cells. The fluorescence of the cells in each well was measured as the total intensity of pixels in the image of a well (Guzman et al., 2014) calculated with background correction (Kalinina et al., 2018). For the validation of the assay, the measurement of the cytotoxicity of control compounds against A549’, VA13, and their derivative cell lines was investigated.

Several cytotoxic and anticancer compounds with different mechanisms of action were selected (Table 1). The experiments were carried out for the cocultures with the FCCT and monocultures with fluorescence detection and with commonly used MTT assays after 72-h incubation of the cells with the investigated compounds. The IC50s (half of the maximal growth inhibition effect) were calculated from the dose–response curves; IC50 estimations were used to simplify the collation with literature data (Supplementary Table S1). The data on cytotoxicity obtained in the both assays are in a good agreement (Table 1).

**TABLE 1:** Cytotoxic action (IC50) of known drugs on A549’, VA13 cell lines, and their derivatives after 72 h of incubation. The cytotoxicity for the cocultures was obtained with the FCCT. For the monocultures, the data were assessed by MTT assays.

Compound	Cytotoxicity for VA13 (IC50, µM)			Cytotoxicity for A549’ (IC50, µM)		
	FCCT (Cocultivation, VA13_Kat and A549’_EGFP)	MTT assay (VA13_Kat)	MTT assay (VA13)	FCCT (Cocultivation, VA13_Kat and A549’_EGFP)	MTT assay (A549’_EGFP)	MTT assay (A549′)
Cycloheximide	0.15 ± 0.09	0.37 ± 0.01	0.37 ± 0.02	0.6 ± 0.3	0.16 ± 0.03	0.25 ± 0.03
Bortezomib	0.0018 ± 0.0005	0.0017 ± 0.0006	0.0011 ± 0.0005	0.0018 ± 0.0004	0.002 ± 0.0001	0.0013 ± 0.0006
Sorafenib	6.1 ± 0.1	3.8 ± 0.2	2.7 ± 1.9	2.1 ± 0.6	2.4 ± 0.3	0.8 ± 0.1
5F-Uracil	22 ± 9	12 ± 4	13.6 ± 0.6	5.5± 2.9	2.8 ± 0.2	4 ± 1
Cisplatin	3.6 ± 0.9	1.54 ± 0.04	2.04 ± 0.08	2 ± 1	1.82 ± 0.04	2.69 ± 0.05
Nocodazole	0.3 ± 0.2	0.3 ± 0.1	0.2 ± 0.1	0.12 ± 0.08	0.26 ± 0.06	0.54 ± 0.27

### High-Throughput Assay for Evaluation of the Selectivity of Cytotoxicity

**FIGURE 2:**
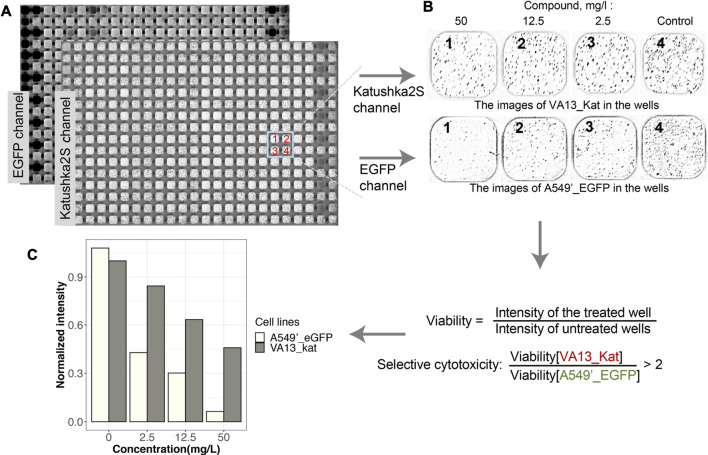
Scheme of analysis of the viability of the cells in mixed culture for the screening of compounds. **(A)**. Scanned images of the plate with cells obtained in the green and far-red channels of fluorescence. The mapped out section of the plate corresponds to three dilutions of one drug (wells 1–3) and a well without a drug (4). Dark pixels in the wells correspond to cells. Light wells without dark pixels in the image indicate the death of the cells after treatment. Uniformly, dark cells on the image correspond to high background levels from fluorescent drugs. **(B)**. Images of cells in the same wells in EGFP and Katushka2S channels are shown after subtraction of the pseudo-background and image fragmentation for viability calculation. **(C)**. An example of the viabilities of cells after treatment with bexarotene and processing of image are given (for details see Methods and Supplementary Figure S4); the selectivity index of cytotoxicity is determined by the ratio of viable VA13_Kat cells to A549’_EGFP.

The FCCT assay was adapted for a high-throughput format in 384-well plates using the autopipette station Janus (for details, see Methods). For each tested compound, four wells per plate with cells growing in cocultures were used; the serial dilutions of a compound were in the first three wells, and the fourth was without treatment. The wells without compounds were used as reference samples and were used to control the uniformity of cell seeding over the plate (Figure 2A, Supplementary Figure S5). The preliminary analyzes were carried out in the concentration range of 2.1–42.5 mg/L. Some of the substances caused the full death of both lines in these concentrations. Therefore, most of the compounds were tested in six dilutions in the range of 0.02–42.5 mg/L. For six serial dilutions, two plates with cells were used.

The effects of compounds on the viability of both cell lines were detected with the scanner after 72 h of incubation. An example of raw images with adjusted brightness and wells with different dilutions of compounds after background subtraction are given in Figure 2. The light wells without dark pixels indicate the death of the cells after treatment (Figures 2A,B). Uniformly, dark images are received for the fluorescent compounds (Figure 2A). Some of them may be used in further analysis after pseudo-background correction (Kalinina et al., 2018) or manual image processing. The image of the whole plate was divided into wells (Figure 2B), and their intensities were calculated (Figure 2C) as described earlier (Guzman et al., 2014). Intensities of the image of wells with medium and without cells were subtracted from intensities of processed images. The intensity of fluorescence in a well normalized the intensity of cells without the addition compounds (Figure 2C, Supplementary Figure S5). The selectivity of cytotoxic action of compounds was assumed in the FCCT if the viability of VA13_Kat cells was two times or higher the survival of the A549’_EGFP line.

### Screening of Selectively Cytotoxic Substances in the Library of Low Molecular Weight Compounds

**FIGURE 3:**
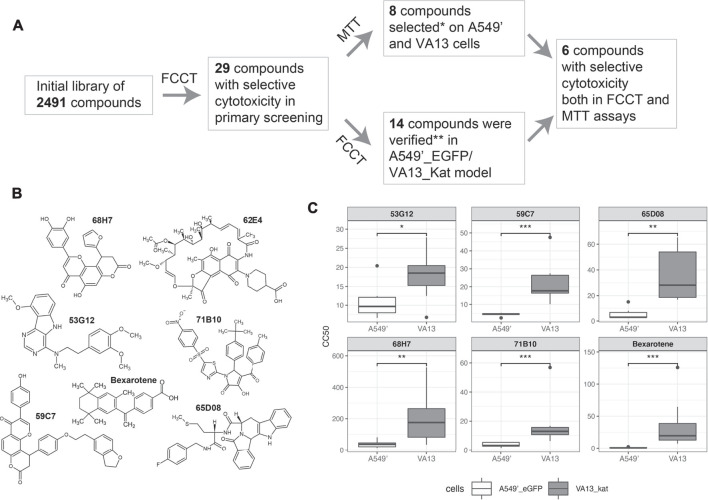
Scheme of the selection of active compounds and their structures. **(A)**. The screening and validation of the selectively cytotoxic compounds. **(B)**. Structures of compounds 59C7, 62E4, 68H7, 71B10, 65D8, and 53G12, that were selectively cytotoxic in both MTT and FCCT tests. *27 compounds from the initial library lot were analyzed in MTT. **28 compounds from a new lot were tested with the FCCT assay. **(C)**. CC50 values of hit compounds were measured in the eight-replicated FCCT test and compared by the Wilcoxon test. Asterisks indicate the range of *p*-values: 0.05 ≤ *p* < 0.1 (*); 0.01 ≤ *p* < 0.05 (**); *p* < 0.01 (***); not significant (NS).

The developed FCCT assay was applied in the HTS format for the detection of selectively cytotoxic substances. For the investigation, we chose the library of 2,491 low molecular weight molecules. These compounds are mostly natural, or their derivatives are with a high diversity of structures (Supplementary Table S2). After screening of the library, the selected compounds were further investigated with the MTT assay and retested in the FCCT as shown in Figure 3A.

In the initial single-replicate FCCT screening, we selected 30 compounds with selective cytotoxicity in two or more consecutive dilutions (Supplementary Table S2, where these compounds are marked as “2”).

To validate the cytotoxic properties of the selected compounds, they were analyzed with the MTT assay on A549′ and VA13 cell lines (Supplementary Table S2). From the obtained dose–response dependences, the concentrations of compounds that caused 50% cell lethality (CC50) were calculated. The compounds were defined as selectively cytotoxic if the difference of CC50 between A549′ and VA13 cells was two times or more. The selectivity of cytotoxicity from initial screening was confirmed for 8 out of 27 tested substances from the leading group. Additionally, these substances were tested with MTT in the breast cancer cell line MCF7 and the cell line of noncancerous origin HEK293T. The compounds were cytotoxic for these cell lines. We observed enhanced toxicities of 65D08, 68H07, and 71B10 in MCF7 in comparison with those of VA13 and lowered in HEK293T cells in comparison with those of A549’ for 71B10 (Supplementary Table S2).

To control the reproducibility of the single-replicate screening, the novel lot of the compounds of the leading group was rescreened in the FCCT. The data were considered to be reproducible if the substance was selective in the FCCT test, at least in one of the concentrations. The selectivity for 14 of 28 tested compounds was detected. Eight of these compounds were not selective in the MTT assay although four of them demonstrated reproducible selectivity in both single-replicate FCCT tests (Supplementary Table S2). Six compounds were confirmed both in MTT and FCCT assays. The chemical structures of these compounds are of high diversity as shown in Figure 3B.

To validate the results of the suggested pipeline, they were retested in eight-replicate FCCT experiments: 1) six leader compounds were confirmed both in MTT and single-replicate FCCT assays and 2) four compounds were selective only in two single-replicate FCCT tests. The CC50s for each replicate were calculated, and the data were compared by the Wilcoxon test (Figure 3C, Supplementary Figure S7). The compound 62E04 was not processed in this way. It is fluorescent, and the classical dose–response curve cannot be built in the FCCT assay of the screen format although selectivity was seen in point dilution. All six leader compounds 59C7, 62E4, 68H7, 71B10, 65D08, and 53G12 and three of four compounds selective only in two single-replicate FCCT tests 66E06, 56E10, and 67D07 (Supplementary Figure S6) were statistically significant and more toxic against cancerous cells.

To validate the fluorescent cell–based assay, fluorescent and nonfluorescent cells were treated with the set of compounds from screening, and control bexarotene and nonfluorescent cells were stained by calcein AM. Besides the calcein AM fluorescence measurement by a plate reader, the viable cells in both assays were imaged with a high-resolution scanner (Supplementary Figure S7). The selectivity in monocultures measured by the fluorescent cell-based assay and calcein AM test (Supplementary Table S3) was significant for six leader compounds validated in the MTT assay (Figure 3), but there was no significant difference for 66E06, 56E10, and 67D07 (Supplementary Figure S6) which were not selective in monocultures in the MTT test, but selective in few independent replicates of the coculture assay.

## Discussion

Cocultures of cells of different origins are widely used for modeling intercellular interactions (for review, see (Miki et al., 2012) and (Jo et al., 2018)). Thus, mixed cultures of cancer and stromal cells were applied for studying the tumor microenvironment (for review, see (Kitaeva et al., 2020)). Cytotoxic effects obtained on cell ensembles may significantly differ from effects detected in monocultures (Alfaro-Moreno et al., 2008). The application of mixed cultures in high-throughput screenings is limited by the complexity of detection and restrictions in reproducibility, cost, and time to set up (Stock et al., 2016). Commonly used assays for cytotoxicity evaluation (for review of methods, see (Ediriweera et al., 2019)) detect the integral signal from the well and cannot distinguish the influence of a treatment on individual cell lines in the mixtures of cells (Dhanya et al., 2019).

Here, we suggest a method for evaluating the selectivity of cytotoxicity in a simple 2D model of lung cancer: Cancerous cell line A549’ was cocultivated with immortalized fibroblasts VA13. The cells were labeled with fluorescent proteins and detected with high-resolution scanning of fluorescence. Thus, it is possible to measure the signal of individual cell lines and to evaluate the selectivity of cytotoxicity of treatment in one well. The adaptation of the method for high-throughput screening simplifies the assessment of the selectivity of cytotoxic effects. This approach allows changing the search criteria in the phenotypic screening for potential anticancer drugs from cytotoxicity to selectivity of cytotoxicity against cancerous cells.

### Selection and Labeling of Cell Lines

Human carcinoma cell line A549’ was selected for the lung tumor model in coculture because it is well studied and has a high proliferation rate, typical for cancer cells. Special attention was paid to the choice of the line of non-tumor etiology because side effects of common chemotherapies are often produced by nonspecifically targeting of dividing healthy cells (Jaffee et al., 2017). Thus, these cells have to divide but grow slower than the cancer ones.

To model normal cells, we had to look for dividing cell lines of the lung origin. The cell line HEK293T is often used as the normal cell model and also used in cocultures (El Debs et al., 2011). However, they are of embryonal kidney origin and grow as fast as A549′ cells (data not shown). In ATCC collection, only the normal lung epithelial cells BEAS-2Bs are available, but they exhibit almost an identical profile of mesenchymal stem cells (Han et al., 2020). Most of the available noncancerous cells from the lung are fibroblasts. They are widely used for the analysis of the effects of the microenvironment (Miki et al., 2012), including cocultures with A549 (Amann et al., 2014). Although primary cultures are more relevant cell models of tissues than long-cultivated cell lines, the proliferation of fibroblasts under conditions of a primary culture is limited, and they may grow even faster than primary carcinoma cells (D. Siemann, 2010). The cell line VA13 of immortalized fibroblasts isolated from a human embryo lung was selected in this work. VA13 cells grow slower than the A549’ cells (Supplementary Figure S2,S3) and are cultivated under the same conditions as the A549’ cells. This imitates accelerated proliferation of tumor cells in comparison with that of normal cells and simplifies cell cultivation.

Selected cell lines were marked by lentivirus integration in the genomes green fluorescent protein EGFP and far-red protein Katuska2S (Figure 1, Supplementary Figure S1) to avoid an overlay of their spectra. The slowly proliferating VA13 cell line was labeled with Katushka2S, which is better for detection in the media in comparison with EGFP (Kalinina et al., 2018); the gene of the last one was integrated into fast-growing A549’. The vice versa labeled cell lines A549’_Kat with Katushka2S and VA13_EGFP with EGFP can be applied (Supplementary Figure S1), but with some reduction in sensitivity of detection with the scanner (data not shown). Other pairs of fluorophores may be used, e.g., EGFP and mCherry (Figure 1C). The random-site integration of fluorescent protein genes by lentivirus may affect adjacent genes and cellular viability. That is why we did not select monoclones and used heterogeneous cell populations to avoid the possible selection of cell clones. Integration of the vector-encoded fluorescent protein did not significantly affect the viability of the cells and does not affect the results of the cytotoxicity test on control drugs as it was proved with MTT (Table 1).

Some compounds, for example, bexarotene (Supplementary Figure S7) or nocodazole (Kalinina et al., 2018) may give the differences in the lower plateau of their dose–response dependencies in comparison with biochemical assays. The observed effect may be caused by the different cellular characteristics of the cells which are detected by both methods. The enzymatic activities in the MTT and calcein AM tests reflect the biochemical activity of the cell, while the fluorescence test indicates cell integrity (Kalinina et al., 2018). In the screenings, applied cell constructs allow investigating cytotoxicity in a monoculture of cells in a high-throughput format. Although exact values may differ, the Pearson correlation was about 0.9 between cytotoxicities calculated in monocultures with the fluorescent cell–based assay and MTT for nonfluorescent compounds in previous work (Kalinina et al., 2018).

### Analysis of the Cytotoxicity of Compounds in a Coculture of Fluorescent Cells

The applicability of the FCCT assay was checked on cytotoxicity of six known drugs. The FCCT data are in a good agreement with the MTT test results on monocultures and literature data (Table 1, Supplementary Table S1). Different parameters of cell survival can explain slight differences in IC50 values: the level of activity of cellular oxidoreductases for the MTT test and the integrity of the cell membrane in the case of the fluorescent protein-based cytotoxicity test (for details see (Kalinina et al., 2018)).

One of the main advantage of cocultivation is the ability to consider possible interactions of the different cell types. Then, mixed culture allows comparing effects on various cell lines on the experimental conditions and simplifies experimental procedures. Thus, for screening of chemicals with unknown solubility, absolute drug concentrations are not anymore critical, since the effect on both lines in one well is compared. Cocultivation of both lines reduces testing costs of the assay. The assay may be applicable not only for selectivity evaluation in drug screening but also for other toxicology applications, e.g., investigations of effects of plasma treatment, where the question is that two cell lines were not cultured in identical media (Semmler et al., 2020) or exposition of cells to nanoparticles (Dasgupta et al., 2016; Ranjan et al., 2020) as one of the viability tests of the cells.

The detection rate may become the limiting stage of high-throughput assays with an application of modern robotic stations. The classical decision for detecting fluorescent cells is using microscopy-based systems, but only the fastest and most expensive of them possess a high detection rate. Thus, we selected a high-resolution scanning-based system (Kalinina et al., 2018). At a lower resolution than microscopy, the scanning is fast enough for reading 384 cells in one detection channel in 15 min; this improves the performance of the method. The fluorescent signal of living cells is detected without disturbing them and adding any reagents, thus allowing the investigation of time-dependent cytotoxicity effects on the same sample of cells.

Fluorescent compounds are difficult for analyzing in the assay where the fluorescent signal is detected. The 16-bit grayscale monochrome image with the 10-µm resolution from a scanner was proceeded to calculate the fluorescence of the cells in each well. The intensity of the cells was below half of the grayscale. The wells with a background intensity of compounds more than three-quarters of the scale were excluded from the analysis automatically or by manual curation. Thus, some compounds accumulating in cells with high fluorescence may be skipped in the screening, although they may have some selectivity as 62E2 (Skvortsov et al., 2020). The wells with fluorescent compounds with an intensity less than the half of the grayscale were processed by the pseudobackground subtraction (Kalinina et al., 2018). The background of the compounds with the intensity up to three-quarters of the scale may be possible to take into account with manual curation by rolling-cycle–based background or pseudo-background subtraction.

The threshold for selective cytotoxicity of a compound was chosen two times of the difference in the corresponding well between the viability of A549’_EGFP and VA13_Kat cells because of the widespread of such threshold in the literature. The criterion of the hit compounds was the selectivity in two sequential dilutions of a compound to select the compounds with selectivity in a wider range of concentrations (Figure 2). First, it was suggested that compounds with selective cytotoxicity in a wider range of concentrations might correlate with a larger “therapeutic window.” Second, in one-repetition screening, the selection of selectively cytotoxic substances in two or more concentrations increases the reliability of the test. Although the accuracy of the fluorescent cell–based assay is comparable with MTT when it is used in replicates (see Table 1, and (Kalinina et al., 2018)), in single-replicate screening, this criterion increases reproducibility.

### Screening of the Library of Structurally Diverse Natural Substances and Their Derivatives

For validation of the suggested FCCT assay and search of selectively cytotoxic compounds, we screened the library of 2,491 compounds. The library consists of structurally diverse natural substances and their derivatives. Although natural compounds are not always the best solution for drug development due to the high complexity of synthesis (Cagan, 2016), they may represent a wider and drug-like chemical space than synthetic derivatives (Harvey et al., 2015). The primary screening reveals the 29 compounds (1.3% of the library), which were further investigated (Figure 3).

The selected compounds were further analyzed with the MTT assay in A549′ and VA13 monocultures. The selectivity of the compound’s action was assumed as confirmed if the difference of СС50 was more than two. Among the 28 studied compounds, nine were selective in A549′ and VA13 cell lines. Additionally, CC50 was measured in the breast cancer cell line MCF7 and fast-growth cell line HEK293T of noncancerous etiology. Only three of these nine compounds were selectively toxic against MCF7 in comparison to those of VA13, and only one was selectively toxic on A549′ in comparison to that of HEK293T. The cytotoxicity of half of the tested substances against all 4 cell lines was in low micromolar concentrations, that is, comparable to that of cisplatin, which was used for the positive control in the MTT test (Supplementary Table S2).

The hit substances were checked in the secondary FCCT screening using new lots from the manufacturer (Figure 3). Half of the compounds (14 of 29 tested) were confirmed in FCCT rescreening, that has good reproducibility for a single-replicate screen. As a result, six selected compounds (0.3% of the library) inhibit A549’ cell growth stronger than those of VA13 in both MTT and FCCT assays (Figure 3).

Few compounds, which were not selective in the MTT assay, e.g. 66B10, 67D07, and 56E10, demonstrated reproducible selectivity in the FCCT in 384-well screens (Supplementary Table S2). Then, they exhibit low or no selective cytotoxicity in individual lines in additional tests in fluorescent cell–based or calcein AM assays (Supplementary Table S3 and Supplementary Figure S7). They appeared to be selectively acting when they were validated under competitive growth conditions in a 96-well low-throughput FCCT format in several replicates (Supplementary Figure S6), although they were with less confidence than the leader compounds. These results confirm that cocultures allow the detection of selective compounds against cancer cells that may be missed in conventional monoculture tests.

The molecules revealed in the screening (Figure 3A, Supplementary Table S2) have diverse structures. These compounds may have some common structural features, such as the indole moiety in 53G12 and 65D08, or the coumarin fragment in 59C7 and 68H7 (Figure 3B). Nevertheless, they have a low Tanimoto similarity score. In addition to diversity, the screening results were quite original. Although some of them are derivatives of well-known classes, e.g. 62E4 is a rifampicin derivative, for most of them, there are no patents or tests related to the antitumor activity of these structures in the open database Pubchem.

Discovered in the present work, molecules are cytotoxic against tumor cells at concentrations more than 1 mg/L and selective in one order of the concentrations range. These properties are too low to consider these drugs as compounds for further direct development into drugs. At the same time, the discovered compounds can be considered as scaffolds for optimization and search for novel structural classes of anticancer drugs. The variety and originality of the structures confirm the effectiveness of the proposed phenotypic screening in the search for substances, which are selectively cytotoxic against cancerous cells.

An increase in the number of hits is possible by in silico enrichment of the libraries by homology with known antitumor compounds or by using the collections of compounds which are directly synthesized with antitumor potential. An increase in the proportion of detected selective compounds and the specificity of their action may further be achieved by the employment of a few different tumor models in the parallel assay.

## Conclusion

The FCCT assay has been developed for the search of substances that are specifically toxic against tumor cells for the primary selection of anticancer drugs. It is based on the cocultivation of A549’_EGFP and VA13_Kat cell lines of tumor and normal etiology, with subsequent detection of the fluorescent signal. The growth of the cells in mixed culture allows taking some of the tumor microenvironment effects into account. The experiment in the same well under equal conditions improves the comparison of the viability of cell lines. The scanning of fluorescence allows detecting cell lines both fast and without disturbing them. The experimental protocol of the FCCT is reduced to three steps: seeding of the cells, addition of the substance, and signal detection.

The method was applied in a high-throughput format for the screening of 2,491 compounds. Six validated with MTT assay compounds were found to be selectively cytotoxic to lung cancer cells A549’, compared with those of the non-tumor lung fibroblasts VA13. Unraveled 4-Hydroxyquinazoline derivatives 56E10 and 67D07 were reproducibly selective in the FCCT although they were invisible in the MTT test with cytotoxicity measurements in individual lines.

## Data Availability

The original contributions presented in the study are included in the article/Supplementary Material. Further inquiries can be directed to the corresponding authors.
