# Supplementary material for: From Toxicity to Selectivity: Coculture of the Fluorescent Tumor and Non-Tumor Lung Cells and High-Throughput Screening of Anticancer Compounds
Source: Front Pharmacol. 2021 Oct 11;12:713103. doi: 10.3389/fphar.2021.713103 (PMC8542663; doi:10.3389/fphar.2021.713103)
Supplement: Supplementary file 1 [file DataSheet1.zip › Table 1.DOCX]

Supplementary Material


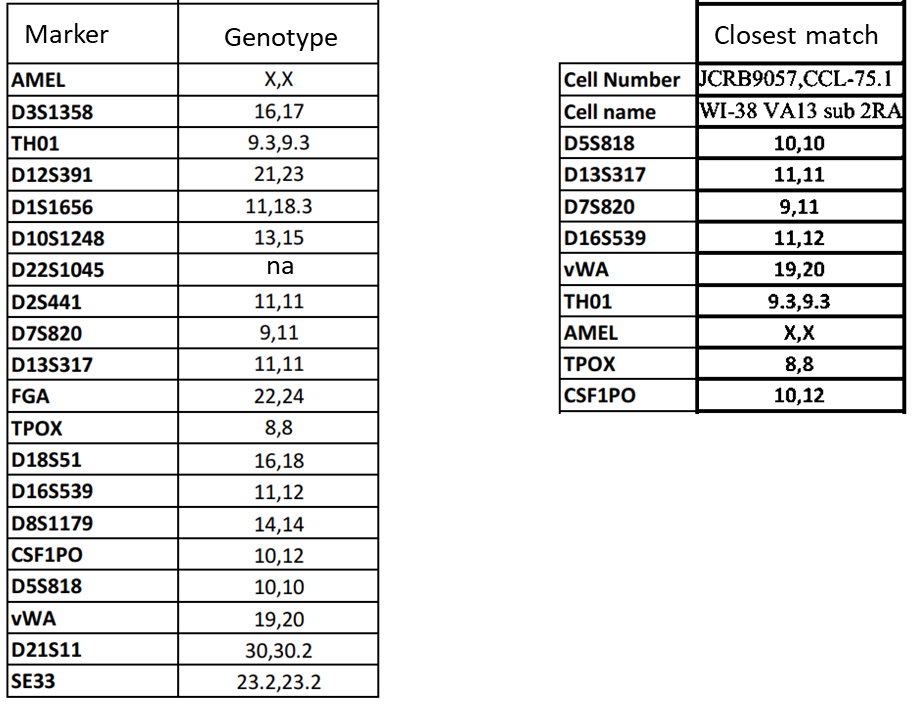
**A.**


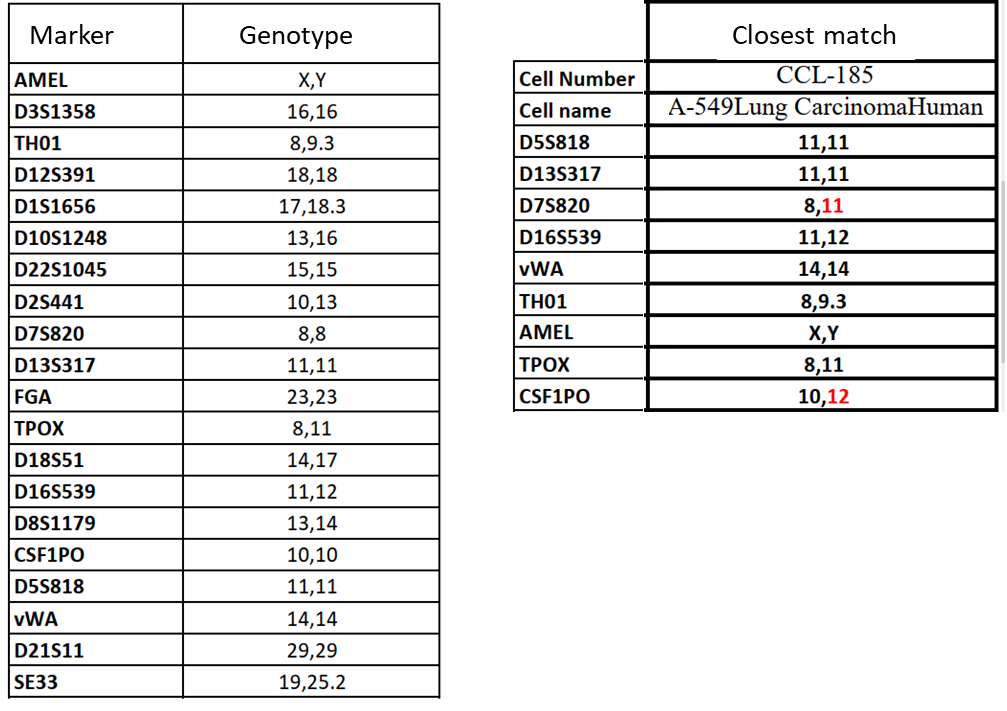
**B.**

**Supplementary Figure 1.** Genotypes of applied cell lines VA13 (**A**) and A549’ (**B**).

**Supplementary Figure 2.** Examples of fluorescent cells in monocultures and co-cultures A. Images of cell lines VA13_Kat used in this work, expressing Katushka2S and A549’_EGFP, expressing EGFP and inverted scheme of labeling cell lines with fluorescent proteins that is based on the cell line A549’_Kat, expressing Katushka2S and VA13_EGFP, expressing EGFP. B. Image of co-cultures of cell lines obtained using the main labeling scheme used in this work (right) and the inverted scheme (left). Images were obtained using an EVOS FL Cell Imaging System fluorescence microscope. EGFP was recorded in the GFP channel (green), Katushka2S - in the RFP channel (red). Scale bar - 200 microns. B. Nuclei are stained with Hoechst and shown in the blue channel. Scale bar - 200 microns.


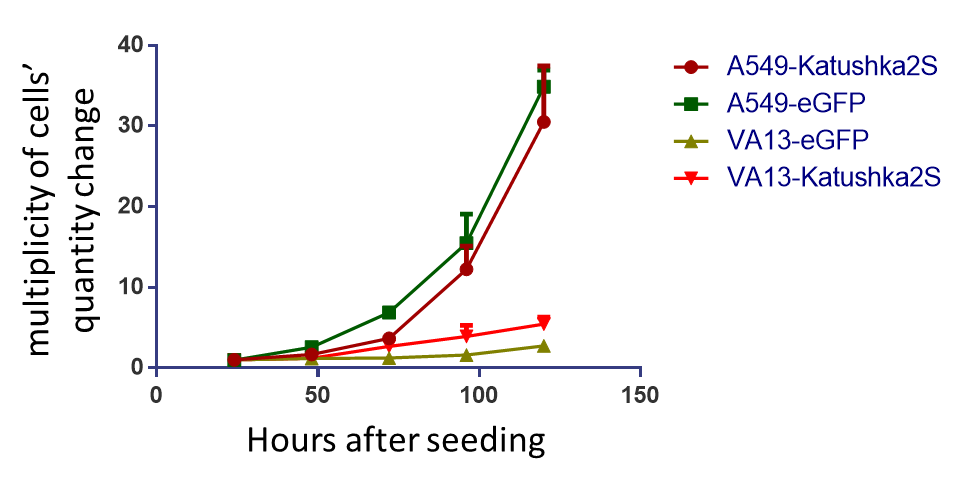


**Supplementary Figure 3.** Dependencies of cell growth from time of fluorescently labeled cell lines A549 and VA13. The amount of A549’ increases in the well of a 48-well plate in 10-15 times, and VA13 in 2-3 times after 96 hours growth. The initial amount of cells was 4 thousand for A549’ derivatives and 8 thousand for VA13 derivatives in a well of a 48-well plate. The number of cells was counted using a Goryaev chamber (3 replicas).


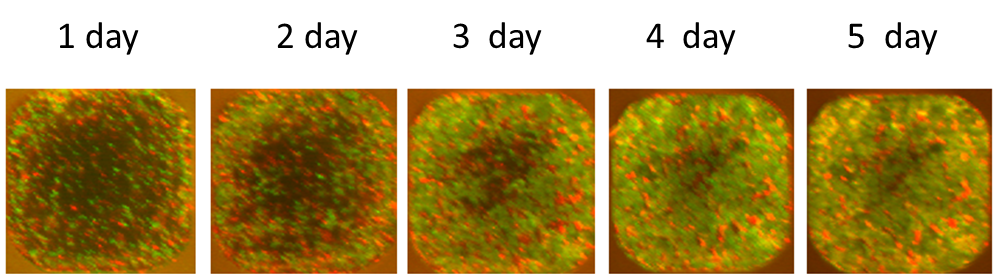


**Supplementary Figure 4.** Growth of the cells in co-culture, imaged with scanner. The images of cell lines A549’-tdTomato (green) and VA13-Katushka2S (red) which were seeded in ratio 1:2 and scanned on TYPHOON since first to five days after plating.

**
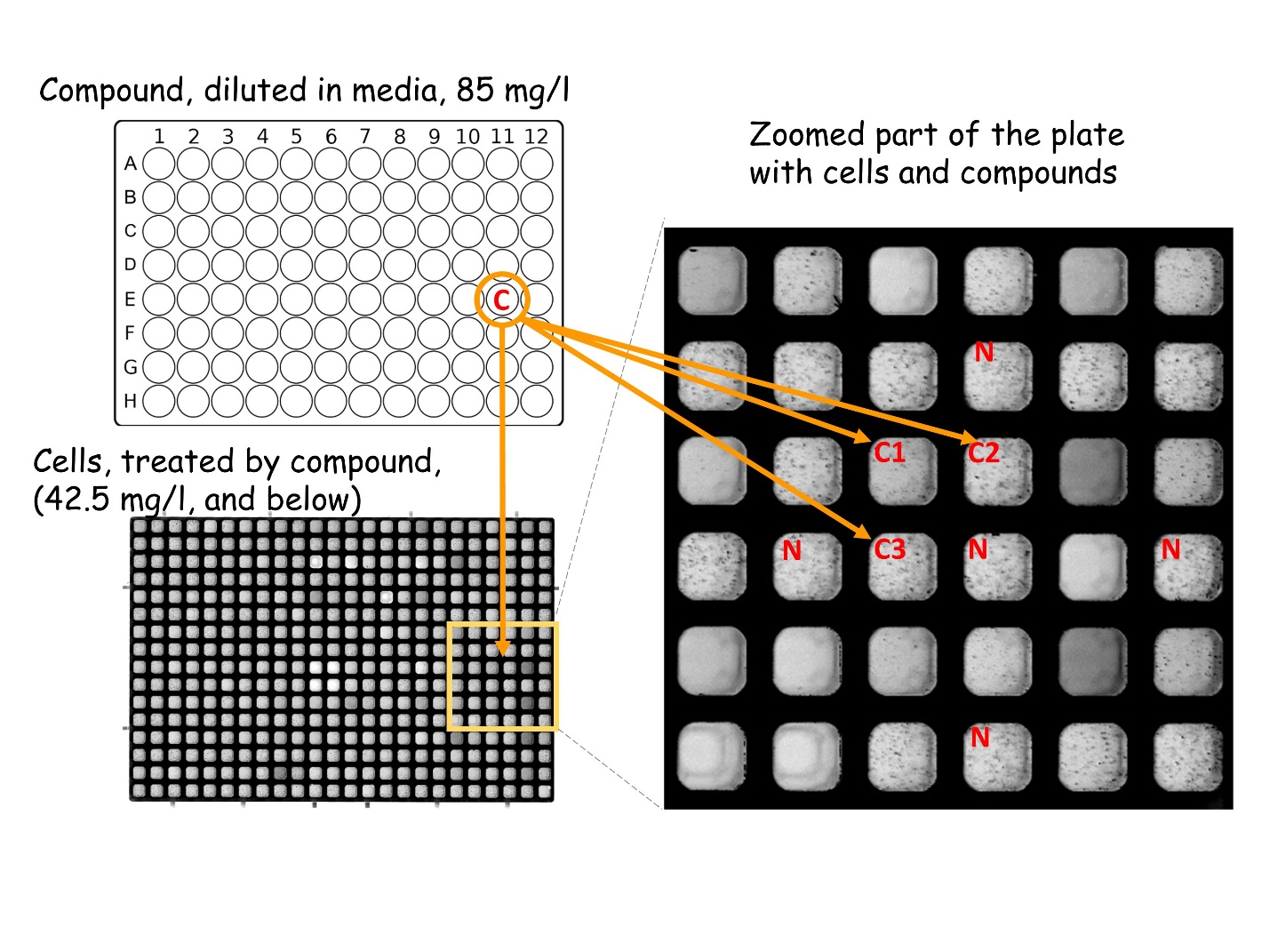
**

**Supplementary Figure 5.** The scheme of location on the plate of the example of the tested compound (C) serially diluted in the wells with cells (C1 = 42.5 mg/L, C2 = 10.6 mg/L, C3 = 2.1 mg/L) and the median of corresponding set of the not treated wells with cells (N) used for normalization


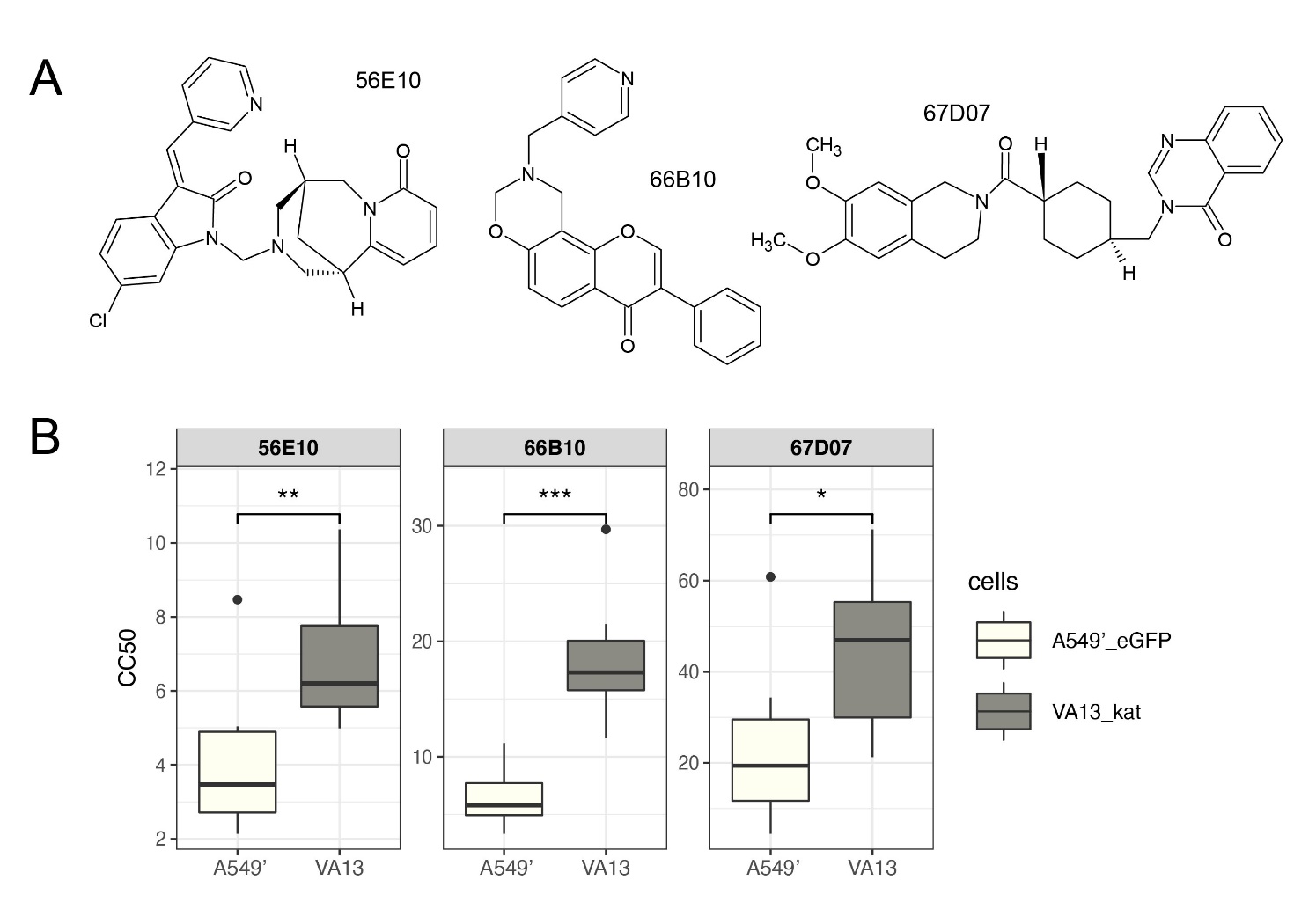


**Supplementary Figure 6.** Three of four compounds selective only in two single-replicate FCCT test 66E06, 56E10, 67D07 were statistically significant more toxic against A549’ cells.

**Supplementary Figure 7.** Images of wells with cells after their treatment with serial dilutions of selected in the screening compounds. Images were acquired with high resolution scanner in EGFP channel for A549’_EGFP cells and CalceinAM treatment of both cell lines and in Katushka channel for VA13_Kat cells and procecced in ImageJ. Each image is representative example of three replicates. (A) Direct fluorescent A549’_EGFP cells detection. (B) CalceinAM staining of A549’ cells. (C) Direct fluorescent VA13_Kat cells detection. (D) CalceinAM staining of VA13 cells.


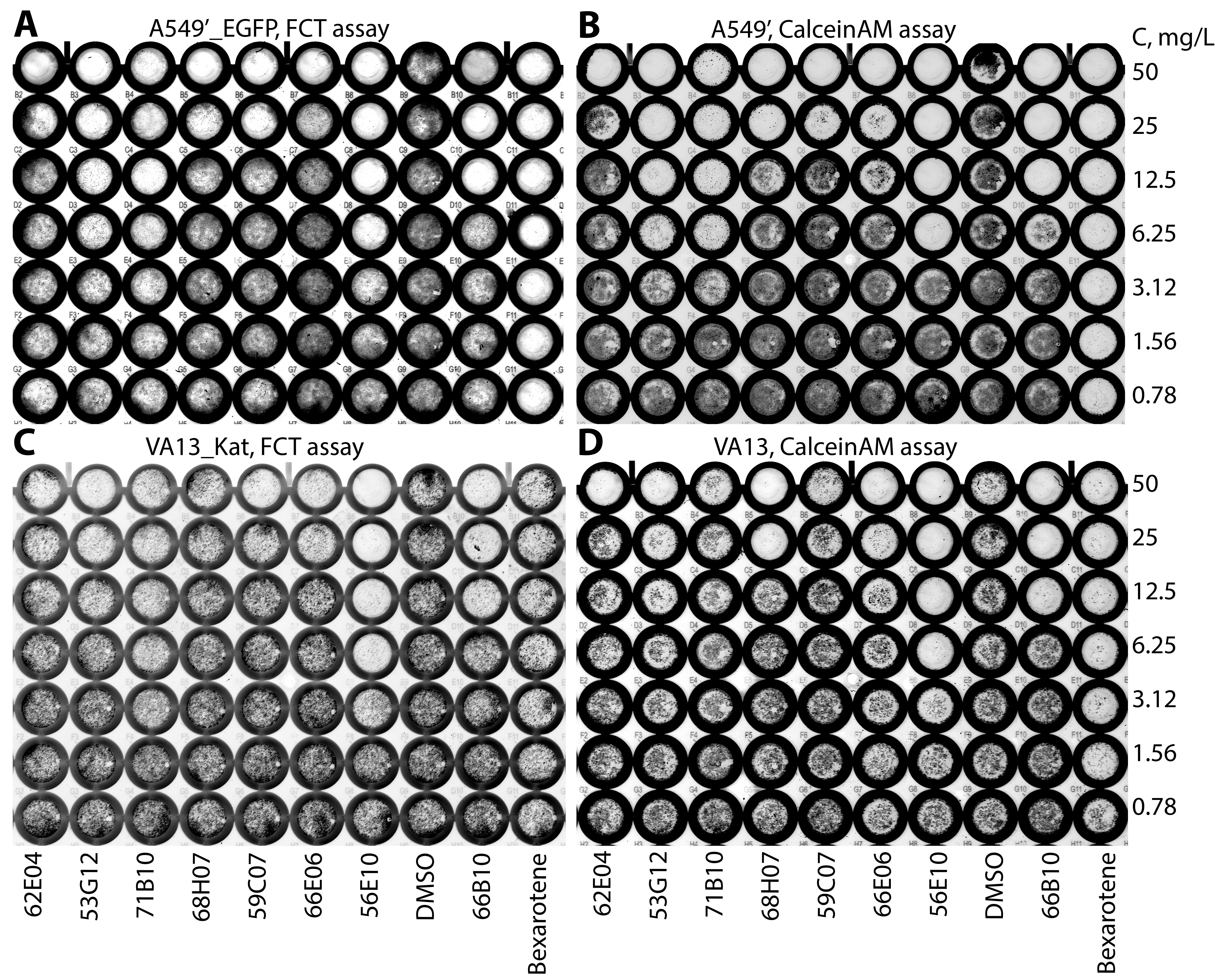


# Supplementary Tables

**Supplementary Table 1.** Cytotoxic action (IC50) of known drugs on A549’, VA13 cell lines, and their derivatives after 72h incubation and corresponding examples of their cytotoxicity from the literature.

**Supplementary Table 2 is in separate file.**

**Supplementary Table 3.** Cytotoxic action (CC50) of compounds measured after 72h incubation in monocultures of fluorescent cells A549’_EGFP, VA13_Kat and in monocultures of noт-fluorescent A549’, VA13 cell lines with fluorescent staining by CalceinAM. “scanning” is image acquisition by high-resolution scanner similar to FCCT assay. “fluometry” is data acquisition by plate fluorimeter in accordance with Calcein AM manufacturer recommendations.

|  | 67D07 | 62E04 | 53G12 | 71B10 | 68H07 | 59C07 | 66E06 | 56E10 | 66B10 | Bexarotene | Doxorubicine |
| --- | --- | --- | --- | --- | --- | --- | --- | --- | --- | --- | --- |
| A549'_EGFP, FCT, scanning | 11.86±0.93 | 28.27±1.07 | 16.56±0.67 | 22.77±4.03 | 27.27±1.97 | 24.97±1.52 | 33.88±1.37 | 6.29±0.63 | 9.6±0.77 | 0.1±0.09 | 15.35±1.13 |
| VA13_Kat, FCT, scanning | 15.83±1.34 | 52.71±7.12 | 24.4±1.2 | 71.26±29.23 | 42.48±1.49 | 41.39±1.77 | 44.19±1.83 | 6.82±0.47 | 16.07±1.6 | na | 32.84±3.81 |
| A549',CalceinAM, scanning | 12.01±1.8 | 25.6±2.84 | 10.17±0.65 | 27.47±5.76 | 20.84±2.24 | 30.25±2.52 | 29.67±1.29 | 3.69±0.18 | 9.84±0.66 | 0.12±0.07 | 9.44±0.73 |
| VA13,CalceinAM, scanning | 14.54±1.9 | 47.72±2.22 | 23.83±1.78 | 58.19±11 | 35.53±2.5 | 46.11±10.12 | 37.07±3.5 | 4.04±0.19 | 13.29±0.98 | 1.75±0.24 | 21.74±2.27 |
| A549',CalceinAM, fluometry | 6.23±0.77 | 34.94±4.47 | 5.44±0.76 | 4.94±0.46 | 14.47±1.33 | 19.16±1.22 | 13.27±1.31 | 3.5±0.27 | 5.25±0.69 | 0.02±0.02 | 2.49±0.29 |
| VA13,CalceinAM, fluometry | 8.53±5.75 | 50.7±7.3 | 21.54±3.06 | 89.27±38.04 | 24.39±1.92 | 30.87±3.94 | 13.34±0.9 | 3.21±0.31 | 8.63±0.88 | 3.53±0.84 | 19.19±4.32 |

# Supplementary References

Guzman, C., Bagga, M., Kaur, A., Westermarck, J., and Abankwa, D. (2014). ColonyArea: an ImageJ plugin to automatically quantify colony formation in clonogenic assays. PLoS One 9, e92444.

Kalinina, M.A., Skvortsov, D.A., Rubtsova, M.P., Komarova, E.S., and Dontsova, O.A. (2018). Cytotoxicity Test Based on Human Cells Labeled with Fluorescent Proteins: Fluorimetry, Photography, and Scanning for High-Throughput Assay. Mol Imaging Biol 20, 368-377.

Mosmann, T. (1983). Rapid colorimetric assay for cellular growth and survival: application to proliferation and cytotoxicity assays. J Immunol Methods 65, 55-63.
